# Motor-Car Cots

**Published:** 1914-06-27

**Authors:** D. D. Kirkaldy

**Affiliations:** Hon. Sec. Nelson Hospital, Wimbledon. St. Abb's, Albany Park Road, Kingston-upon-Thames


*To the Editor of* The Hospital.

### Motor-Car Cots.

*To the Editor of* The Hospital.

Sir,—The suggestion you were good enough to make in your issue of June 6 that the Nelson Hospital should endeavour to raise funds for "Motor-Car Cots" was brought before the Council on the Thursday following.

It was, however, resolved that the most that could be done was to appeal for donations to owners of cars concerned in accidents brought into the hospital, and that this should be done when owners were traced. The chief difficulty which presents itself to me with regard to appealing to motor-car owners to found such cots is that it would be almost impossible to prevent the owners inferring that the appeals insinuated that they were careless of endangering life and limb, and should therefore make provision for the probable results of their carelessness by having beds ready for their victims—such of them, at least, as were not killed outright—and thus turning them at once against the objects appealed for.

I trust that, through the resolution mentioned above, some help may come to the hospital from the suggestion you have kindly put forward.

I am, dear Sir, yours faithfully,
D. D. Kirkaldy, Hon. Sec.
Nelson Hospital, Wimbledon.

St. Abb's, Albany Park Road,
Kingston-upon-Thames, June 16, 1914.

[We are glad to think that our suggestion has at least had the result of a decision to approach more carefully than before owners of cars concerned in accidents brought to the hospital. We think, however, that Mr. Kirkaldy mistakes the impression which an appeal to motor-car owners generally would make. The driver who waits is much more inclined after an accident to take the attitude of an injured party. The average motorist knows quite well that the dangers of our streets spring rather from a mixed traffic than from car-drivers' carelessness; and recent statistics have shown that the bicycle is one of the most prolific occasions of accidents. The child at play in the road, the dog nosing in the gutter, these, in a state of mixed traffic, are the occasions of numerous accidents to-day. There would, we believe therefore, be little difficulty in convincing the average motor-car owner that the one thing he needs to clinch his case against our present street chaos is a voluntary effort to provide for those accidents, the risk of which is a constant anxiety to him.—Ed. The Hospital.]

